# Dimensionality reduction reveals fine-scale structure in the Japanese population with consequences for polygenic risk prediction

**DOI:** 10.1038/s41467-020-15194-z

**Published:** 2020-03-26

**Authors:** Saori Sakaue, Jun Hirata, Masahiro Kanai, Ken Suzuki, Masato Akiyama, Chun Lai Too, Thurayya Arayssi, Mohammed Hammoudeh, Samar Al Emadi, Basel K. Masri, Hussein Halabi, Humeira Badsha, Imad W. Uthman, Richa Saxena, Leonid Padyukov, Makoto Hirata, Koichi Matsuda, Yoshinori Murakami, Yoichiro Kamatani, Yukinori Okada

**Affiliations:** 1 Department of Statistical Genetics, Osaka University Graduate School of Medicine, Suita, 565-0871 Japan; 2 Laboratory for Statistical Analysis, RIKEN Center for Integrative Medical Sciences, Yokohama, 230-0045 Japan; 3 Department of Allergy and Rheumatology, Graduate School of Medicine, the University of Tokyo, Tokyo, 113-8655 Japan; 4 Pharmaceutical Discovery Research Laboratories, TEIJIN PHARMA LIMITED, Hino, 191-8512 Japan; 5 Department of Biomedical Informatics, Harvard Medical School, Boston, MA 02115 USA; 6 Department of Ophthalmology, Graduate School of Medical Sciences, Kyushu University, Fukuoka, Fukuoka, 812-8582 Japan; 7 Allergy and Immunology Research Center, Institute for Medical Research, Ministry of Health Malaysia, 40170 Setia Alam, Malaysia; 8 Division of Rheumatology, Department of Medicine, Karolinska Institutet and Karolinska University Hospital, 17177 Stockholm, Sweden; 9 Department of Internal Medicine, Weill Cornell Medicine-Qatar, Education City, Doha, 24144 Qatar; 10 Department of Internal Medicine, Hamad Medical Corporation, Doha, 3050 Qatar; 11 Department of Internal Medicine, Jordan Hospital, Amman, 520248 Jordan; 12 Rheumatology Division, Department of Internal Medicine, King Faisal Specialist Hospital and Research Center, Jeddah, H45X+P6 Saudi Arabia; 13 Dr. Humeira Badsha Medical Center, Emirates Hospital, Dubai, 391203 United Arab Emirates; 14 Department of Rheumatology, American University of Beirut, Beirut, 11-0236 Lebanon; 15 Center for Genomic Medicine, Department of Anesthesia, Critical Care and Pain Medicine, Massachusetts General Hospital, Harvard Medical School, Boston, MA 02115 USA; 16 Program in Medical and Population Genetics Broad Institute, Cambridge, MA 02142 USA; 17 Laboratory of Genome Technology, Institute of Medical Science, the University of Tokyo, Tokyo, 108-8639 Japan; 18 Department of Computational Biology and Medical Sciences, Graduate school of Frontier Sciences, the University of Tokyo, Tokyo, 108-8639 Japan; 19 Division of Molecular Pathology, Institute of Medical Science, the University of Tokyo, Tokyo, 108-8639 Japan; 20 Laboratory of Complex Trait Genomics, Department of Computational Biology and Medical Sciences, Graduate School of Frontier Sciences, the University of Tokyo, Tokyo, 108-8639 Japan; 21 Laboratory of Statistical Immunology, Immunology Frontier Research Center (WPI-IFReC), Osaka University, Suita, 565-0871 Japan; 22 Integrated Frontier Research for Medical Science Division, Institute for Open and Transdisciplinary Research Initiatives, Osaka University, Suita, 565-0871 Japan

**Keywords:** Genome informatics, Genetic association study, Genetic variation, Risk factors

## Abstract

The diversity in our genome is crucial to understanding the demographic history of worldwide populations. However, we have yet to know whether subtle genetic differences within a population can be disentangled, or whether they have an impact on complex traits. Here we apply dimensionality reduction methods (PCA, *t*-SNE, PCA-*t*-SNE, UMAP, and PCA-UMAP) to biobank-derived genomic data of a Japanese population (*n* = 169,719). Dimensionality reduction reveals fine-scale population structure, conspicuously differentiating adjacent insular subpopulations. We further enluciate the demographic landscape of these Japanese subpopulations using population genetics analyses. Finally, we perform phenome-wide polygenic risk score (PRS) analyses on 67 complex traits. Differences in PRS between the deconvoluted subpopulations are not always concordant with those in the observed phenotypes, suggesting that the PRS differences might reflect biases from the uncorrected structure, in a trait-dependent manner. This study suggests that such an uncorrected structure can be a potential pitfall in the clinical application of PRS.

## Abstract (web summary)

Population structure, even subtle differences within seemingly homogenous populations, can have an impact on the accuracy of polygenic prediction. Here, Sakaue et al. use dimensionality reduction methods to reveal fine-scale structure in the Biobank Japan cohort and explore the performance of polygenic risk scores.

## Introduction

We humans are unprecedentedly thriving mammals in the long history of life on earth. Anatomically modern humans migrated out of Africa^1^, outlived all other hominins^2^, and spread around the globe in a surprisingly short period. We now live from high mountains to deep forests, from vast deserts in continents to tiny islands in the ocean. The history of migration, admixture, and adaptation shapes the diversity recorded in the human genome. The ever-expanding knowledge from genomic analysis of worldwide populations revealed the differences that exist among us. People living in extreme environments demonstrate how humans can be genetically adapted to the cold^3^, high altitude^4,5^, or diving into the sea^6^. However, we do not yet know to what degree our genomes differ due to subtle environmental and regional diversity. We have little knowledge about whether neighboring two regions are different just culturally and linguistically, or also genetically.

We can decipher our origin and diversity through genomic differences. Since the genomic differences among individuals are millions in dimension, we need a practical method to reduce this dimension and render it understandable for humans. Principal component analysis (PCA), a classical dimensionality reduction method, has been a method of choice to uncover the large population structure^7,8^. This linear transformation, however, was not sufficient to fully capture the fine and subtle genomic structure. Additional non-linear dimensionality reduction methods, *t*-distributed stochastic neighbor embedding (*t*-SNE) and its combination with PCA (PCA–*t*-SNE), were applied, and exhibited the ability to provide interpretations of complex population structures and disease biology^9–11^. Recently, uniform manifold approximation and projection (UMAP) was developed as a novel non-linear dimensionality reduction method capable of clearly distinguishing neighboring clusters while retaining the global structure. UMAP and its combination with PCA (PCA–UMAP) are also computationally fast and scalable for application to large genomic datasets^12^. These novel dimensionality reduction methods should be applied to the relatively understudied diverse populations worldwide to uncover the unknown fine-scale structure.

One of the overriding tasks of genomic study is to improve healthcare through accurate genetic prediction. While this effort clearly requires genomic knowledge of worldwide populations, the current genomic studies are largely biased toward the Western continental populations^13^. Determining the subtle structural differences of diverse ancestries, such as neighboring regions or islands within a population, would be critical to avoid future health disparities, because these genomic structures within a population have unexpectedly been raised as potential confounding factors in the clinical prediction of health risks^14^. Most notably, much debate exists on (i) how such genomic structures would be reflected on the estimation of the polygenic risk score (PRS) of complex human traits and (ii) whether such cryptic structures in PRS can be corrected.

Japan, located far east of Africa and Europe as one of the ends of the human journey, has experienced a unique demographic history. One hypothesis assumes that two waves of human migration into Japan occurred: one from Southeast Asia 40,000 years ago, followed by another from the Korean Peninsula 3000 years ago^15,16^. Few admixture events have taken place after these migratory waves, and the population has been kept isolated within the mainland and the surrounding thousands of small islands. These unique situations represent an ideal scenario for the investigation of the fine-scale structure of neighboring yet isolated regions, which might be in contrast to admixed populations living on a continent.

**Fig. 1:**
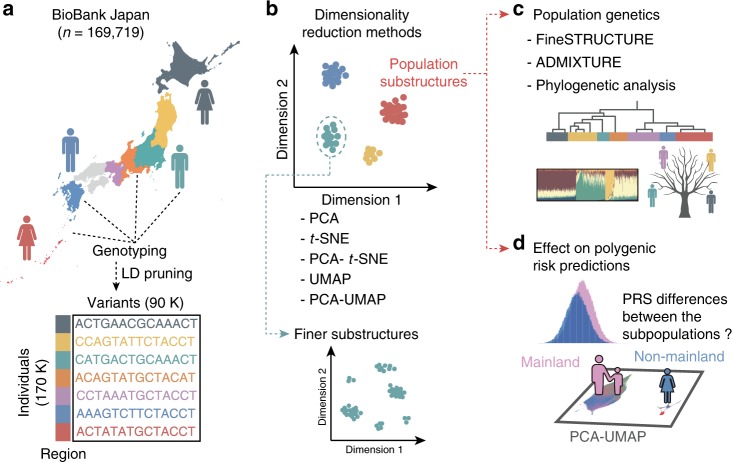
Overview of this study. **a** Japanese individuals from seven major regions of Japan were genotyped. Phased and imputed genotypes were linkage disequilibrium (LD)-pruned and formatted as an input for dimensionality reduction methods. **b** We applied five dimensionality reduction methods to the genotype. We further applied PCA–UMAP to the subpopulations in an attempt to identify even finer substructures. **c** We performed fineSTRUCTURE, ADMIXTURE, and phylogenetic analyses in each subpopulation identified in **b**. **d** We investigated how the identified subpopulations affected polygenic risk predictions in a phenome-wide scale.

Here, we comprehensively apply a series of linear and non-linear dimensionality reduction methods (PCA, *t*-SNE, PCA–*t*-SNE, UMAP, and PCA–UMAP) to large-scale biobank-derived genomic data in the Japanese population (*n* > 170,000), to deconvolute the genetic differences within this population (Fig. 1a, b). We further aim to interpret the identified subpopulations using other population genetics methods (Fig. 1c). Finally, we quantify the differences in PRS related to the subtle population structures (Fig. 1d). In a phenotype-dependent manner, our results demonstrate that such biases in PRS would not be fully correctable even with pre-detection of the cryptic structures.

## Results

### Dimensionality reduction methods to the Japanese population

Japan consists of more than 6000 islands located around the mainland. The population has experienced few admixture events after the two waves of human migration from the Eurasian Continent^17^. Separated by the sea, the Japanese people have been recognized as a demographically homogeneous and isolated population. However, there exist diverse cultural and linguistic differences from region to region and from island to island even within the Japanese population. To uncover the finest-scale structure within the Japanese population, we first applied five dimensionality reduction methods to the large-scale Japanese genotype data (*n* = 169,719): (i) PCA, an orthogonal linear transformation which projects the genotype data into the new reduced dimensional space such that the greater variance comes in an order; (ii) *t*-SNE, a non-linear dimensionality reduction method that converts similarities between data points to joint probabilities and minimizes the Kullback–Leibler divergence between the joint probabilities of the low-dimensional embedding and the high-dimensional data^18^; (iii) PCA–*t*-SNE, an application of *t*-SNE for principal components of genotype data; (iv) UMAP, a novel non-linear dimensionality reduction technique based on Riemannian geometry and algebraic topology to model and preserve the high-dimensional topology of data points in the low-dimensional space; and (v) PCA–UMAP, an application of UMAP for principal components of genotype data to be computationally more advantageous and statistically less noisy (PCA–UMAP)^12,19^.

**Fig. 2:**
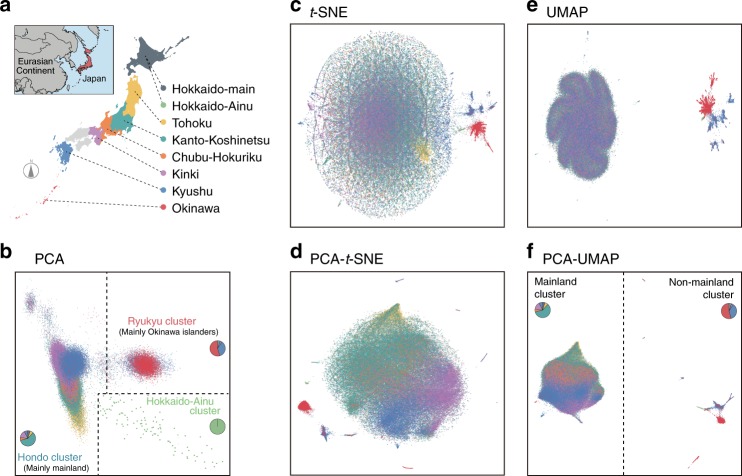
Dimensionality reduction of biobank-scale genotype data from the Japanese population. Two-dimensional illustrations of biobank-scale genotype data from the Japanese population by the five dimensionality reduction methods. The color of individual points indicates the region where a given study individual was recruited. **a** Geographic description of the Japanese islands and the definitions of the regions and colors. **b** The first two principal components from PCA. Individuals in Hondo (mainly in the mainland), in Ryukyu (mainly in Okinawa and surrounding islands), and in Hokkaido-Ainu (indigenous population in Hokkaido) were defined as described previously. **c–e** Two-dimensional illustrations by **c** *t*-SNE, **d** PCA-*t*-SNE, **e** UMAP, and **f** PCA–UMAP. Individuals in the mainland and non-mainland clusters were defined based on the PCA–UMAP results. The pie charts depicted in **b**, **f** represent the constitutions of individuals, who were marked according to the recruitment regions in corresponding colors.

The genetic encoding of the 91,551 variants, which were pruned based on the linkage disequilibrium (LD) structure, was deconvoluted down for illustration in a two-dimensional space (Fig. 2). The distribution of the individuals projected onto the two-dimensional space by PCA largely reflected the geographic distribution. The PC1 corresponded to the northeast-to-southwest geographic axis, which separated the Japanese population into two major clusters, as reported previously^16,20^. The “Hondo” cluster consisted mainly of the residents of the mainland (the four major Japanese islands, which included six regions in this study [i.e., Hokkaido, Tohoku, Kanto-Koshinetsu, Chubu-Hokuriku, Kinki, and Kyushu]; Supplementary Fig. 1). The “Ryukyu” cluster consisted mainly of the residents of Okinawa and part of Kyushu. Additionally, a small clear outlier cluster from the two large clusters was observed, as highlighted in green in Fig. 2b. As the individuals within this cluster were recruited mostly from the Hokkaido region, they are termed the “Hokkaido-Ainu” population, henceforth; the “Ainu” is the Japanese indigenous population of the northernmost island, which has undergone a different demographic history from the mainland population as described previously^15^.

Among the five methods of dimensionality reduction, UMAP and PCA–UMAP clearly and discretely distinguished individuals in the mainland from those in the surrounding islands and Hokkaido-Ainu district (termed the “non-mainland” population henceforth). Conversely, the continuous or juxtaposed distribution of individuals observed in PCA, *t*-SNE, and PCA–*t*-SNE made it difficult to distinguish them without ambiguity. In PCA–UMAP, even the northeast-to-southwest structure within the mainland could be observed correctly compared with the UMAP approach, in which the structure within the mainland was not maintained. To validate the two large subclusters (i.e., mainland and non-mainland) identified by PCA–UMAP, we compared the classification based on the subclusters with the individual-level regional information of the recruitment centers. The degree of correspondence, as measured using a metric Cohen’s kappa^21^, was almost perfect (kappa = 0.821, *P* < 1 × 10^−300^)^22^. We also noted that PCA–UMAP was capable of discretely distinguishing the Japanese population from other East Asian populations in 1000 Genomes Project (1KGP)^23^, as described for the global populations^12^ (Supplementary Fig. 2).

### PCA–UMAP revealed population substructures within Japan

**Fig. 3:**
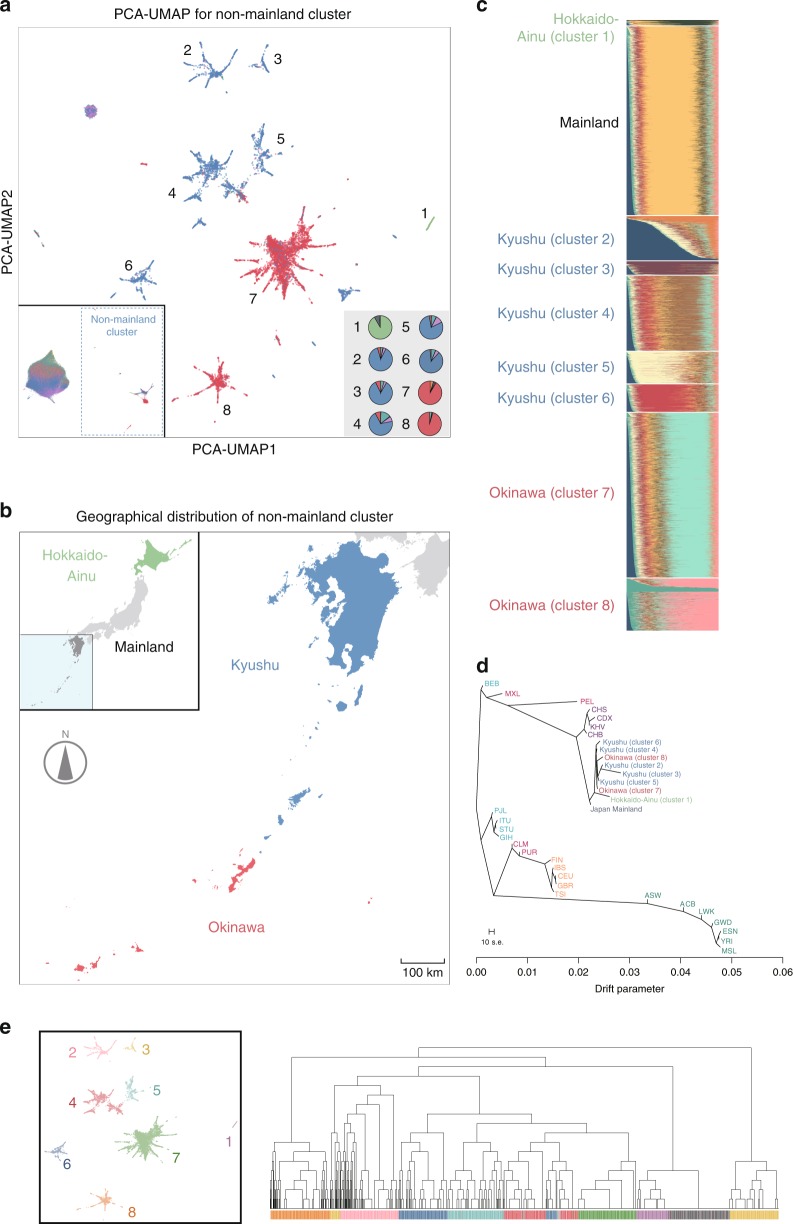
Fine-scale population structure disentangled by PCA–UMAP, and its validation using population genetics methods. **a** Secondary PCA–UMAP to individuals within the non-mainland cluster. The color of individual points indicates the region from which a given study individual was recruited, as shown in Fig. 2. The numbers (1–8) in the main figure represent the subcluster definition, which is described in detail in Supplementary Fig. 4. The bottom-left inset shows the results of PCA–UMAP to all the individuals in the cohort, and the pie charts in the bottom-right inset represent the constitutions of the subcluster individuals annotated according to the recruitment regions in corresponding colors. **b** Geographic and color descriptions of the regions shown in **a**. The inset describes the Japanese islands, and the main panel describes the expanded view of the southwest islands of the Ryukyu region of Japan (regions colored in blue in the inset). **c** ADMIXTURE analysis using the unsupervised maximum-likelihood method under a model with 11 ancestral components (*k* *=* 11). **d** Maximum-likelihood phylogenetic tree of the Japanese subpopulations defined in Fig. 3a and of the worldwide populations from the 1KGP. The scale bar shows the average standard error of the entries in the covariance matrix. **e** Correspondence between the secondary PCA–UMAP to the non-mainland cluster and the hierarchical clustering performed by using fineSTRUCTURE. The right panel shows the clustering results of fineSTRUCTURE, in which individuals are annotated and colored according to the subclusters defined by PCA–UMAP (left panel).

Motivated by the ability of PCA–UMAP to better identify the fine-scale structure of the Japanese population compared with the other four methods, we sought to evaluate whether PCA–UMAP can further separate insular subpopulations, despite their geographic adjacency. We applied PCA–UMAP to the genotypes of individuals who were categorized in the mainland or non-mainland clusters in the primary PCA–UMAP analysis. While PCA–UMAP of the mainland cluster did not yield further dissociation (Supplementary Fig. 3), the application of PCA–UMAP to the non-mainland cluster dissolved it into eight different subclusters (Fig. 3a and Supplementary Fig. 4 for cluster definition). One of the subclusters was consisted mostly of the northernmost Hokkaido-Ainu cluster (cluster 1), whereas the remaining seven (clusters 2–8) were subpopulations from southwest islands located in the Ryukyu region of Japan (Kyushu and Okinawa, Fig. 3b). These southwest islands are located adjacently, as close as 50 km apart from each other, and had never been depicted as genetically distinct clusters in the previous dimensionality reduction methods.

Projection on the transitional layers allows tracking individual correspondence across each of the five dimensionality reduction methods (Supplementary Fig. 5). In this layered illustration, we observed several instances in which individuals in the non-mainland population fell into the largest cluster (i.e., the mainland cluster) when using methods other than PCA–UMAP (red arrows in Supplementary Fig. 5).

To validate these subclusters, we merged clusters 2–6 as “Kyushu subcluster” and clusters 7 and 8 as “Okinawa subcluster.” Then, we measured the correspondence between merged subclusters by PCA–UMAP and individual-level regional information of recruitment centers. We observed a high level of correspondence between them (kappa = 0.821, *P* < 1 × 10^−300^).

To gain more insights into the finest-scale substructures (i.e., subclusters 1–8) identified by PCA–UMAP, we applied the recently developed method, fineSTRUCTURE^24,25^, which can segregate individuals into genetic clusters hierarchically by taking individual haplotype information. We applied fineSTRUCTURE to randomly selected subsets of mainland individuals (*n* = 200) and non-mainland individuals (*n* = 1508). The hierarchical clustering corresponded mostly to the subclusters identified by PCA–UMAP, although there were some differences caused by the level of coarseness or fineness of the clustering (Fig. 3e). If we defined manually the correspondence at the level of 16 clusters as shown in Supplementary Fig. 6d, the concordance was strikingly high (kappa = 0.954, *P* < 1 × 10^−300^). We considered that the substructures identified by PCA–UMAP (genotype-based) were concordant with those identified by fineSTRUCTURE (haplotype-based).

To investigate the origin and history of the finest-scale subpopulations identified by PCA–UMAP, we applied a set of analytical methods of population genetics. First, we sought to separate the genotype data into discrete ancestral components, to capture the genetic history of the structured population^26^. To this end, we performed an ADMIXTURE analysis in randomly selected mainland individuals (*n* = 3000) and the non-mainland individuals identified by PCA–UMAP, with *k* (the number of assumed ancestral components) ranging from 2 to 14. The ADMIXTURE analysis at *k* ≥ 11 revealed that the prominent genetic components were specific to each of the subclusters, even though they were located adjacently (Fig. 3c, full result in Supplementary Fig. 7a). In accordance with the PCA–UMAP results, at *k* = 2 or 3, we also observed that the different genetic components (light green and dark green in Supplementary Fig. 7a) characterized the mainland and non-mainland populations. We did not observe an apparent overlap of the genetic components between Japanese and other East Asians from the 1KGP (Supplementary Fig. 7b). Second, we inferred the patterns of population splits and mixtures based on the phylogenetic analysis^27^. The allele frequency data of individuals within the subclusters, of those in the mainland of Japan, and of worldwide populations from the 1KGP, were phylogenetically analyzed using Treemix software. Recapitulating the known path of the global population history, the Japanese population diverged first from the South Asian population, followed by the separation from the Chinese population (Fig. 3d). Intriguingly, we next observed the divergence of the Japanese mainland population from Hokkaido-Ainu (cluster 1) and southwest insular subpopulations (clusters 2–8), which were both identified as a non-mainland cluster by PCA–UMAP. This observation is consistent with the hypothetical demographic history, which proposes that the first wave of human migration from the Eurasian Continent to Japan was the origin of the individuals in Hokkaido-Ainu and southwest islands (i.e., non-mainland population), even though those two subpopulations are now located more than 3000 km apart geographically. Taken together, the results of PCA–UMAP and population genetics tools shed light on the diverse genomic history of Japan.

### Dimensionality reduction methods to worldwide populations

**Fig. 4:**
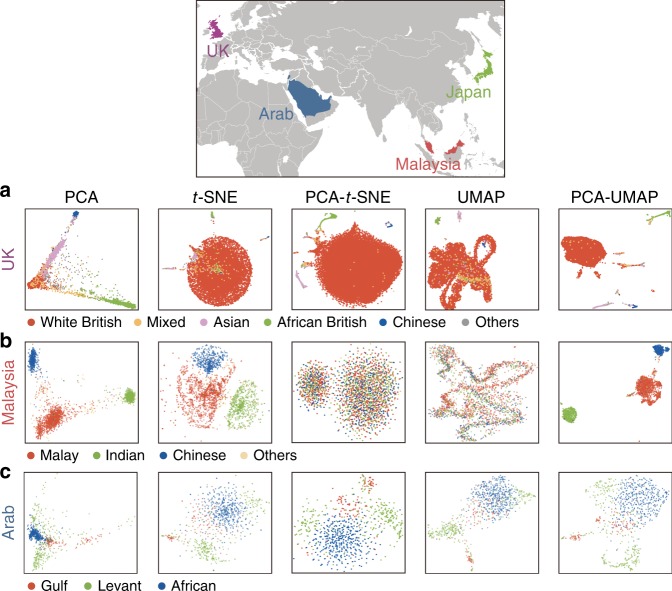
Application of dimensionality reduction methods to worldwide populations. Results of the application of the five dimensionality reduction methods to genotypes from **a** the United Kingdom (UK), **b** Malaysia, and **c** the Arab population. In each of the cohorts, an individual plot is annotated by colors indicating the self-reported ancestry recorded in the cohort.

We next sought to investigate the generalizability of the performance of the dimensionality reduction methods observed in the Japanese insular population. Notably, given the importance of the demographic history and the degree of admixture on the genomic population structures, the performance should be tested in a broader context including relatively understudied populations. We thus applied the five dimensionality reduction methods to the genotype data from (i) UK Biobank (subset, *n* = 54,293; see “Methods” section)^28^, (ii) the Malaysian Epidemiological Investigation of Rheumatoid Arthritis (MyEIRA; Malaysian cohort; *n* = 2831)^29^, and (iii) the Genetics of Rheumatoid Arthritis in Some Arab States Study (Arab cohort; *n* = 863)^30^. First, UK Biobank results suggested that PCA–UMAP could most discretely and finely distinguish subpopulations other than those with white British ancestry (red large cluster in Fig. 4a). Second, the results from the MyEIRA study showed that PCA–UMAP could again conspicuously identify the subpopulations within the Malaysian population discretely, which was strikingly concordant with self-reported ancestry (Fig. 4b). The population clusters from PCA–UMAP were quite apparent in the Malaysian population, in which we speculate that few admixture events occurred among the ancestries. Third, in Arab cohort, although we observed population substructures, it was difficult to define subpopulations discretely (Fig. 4c), which was in contrast with the previous examples. In addition, the concordance with the self-reported ancestry was lower than it was in the previous examples. Given the demographic history of the Arab population, we considered the possibility that the degree of admixture among the ancestries within the Arab cohort was high, which made it difficult to divide the cohort into discrete clusters. Taken together, we concluded that PCA–UMAP generally had the capacity to identify discrete subclusters within a population, if the degree of admixture was not high across the subclusters.

### Population substructures can cause a bias in PRS prediction

One of the goals of genomic research is the improvement of healthcare through individualized medicine. With an exponential increase in genetic studies in terms of number and power, PRS is poised to predict the inborn health risks. Trans-ethnic collaborative works also showed the utility of PRS in genetics-driven identification of key drivers of health outcomes^31^. However, a considerable debate on how to implement PRS into clinical practice remains, particularly regarding its susceptibility to biases. These biases have been described to be potentially caused by cryptic population structures^32^. Nevertheless, the previous studies of these biases have focused mainly on the large-scale structure of a population with European ancestry, and were limited in the number of the investigated traits.

Given the enthusiasm on the upcoming clinical application of PRS, we need to address the potential biases finely and comprehensively including non-European populations. Harnessing our demonstration of the finest-scale population structure within Japan, we finally investigated the PRS differences among the subpopulations. Since the genetic architecture is different according to trait^33^, the PRS differences need to be assessed on a spectrum of complex human traits. To this end, we performed GWASs of 45 quantitative traits on the randomly chosen half of the individuals in the Japanese cohort as a discovery group. The phenotypes were adjusted for sex, age, age^2^, 20 principal components, and a binary variable indicating whether an individual was identified as a member of the mainland or non-mainland cluster based on the result of PCA–UMAP, to account for as much potential confounders as possible. Then, we calculated PRSs using the GWAS statistics by a thresholding and clumping method in the other half of individuals as a validation group. We thus obtained the normalized PRSs and the normalized phenotypic values, and quantified the differences between the values of the non-mainlanders and those of the mainlanders (=*Δ* normalized PRS and *Δ* normalized phenotypic value).

**Fig. 5:**
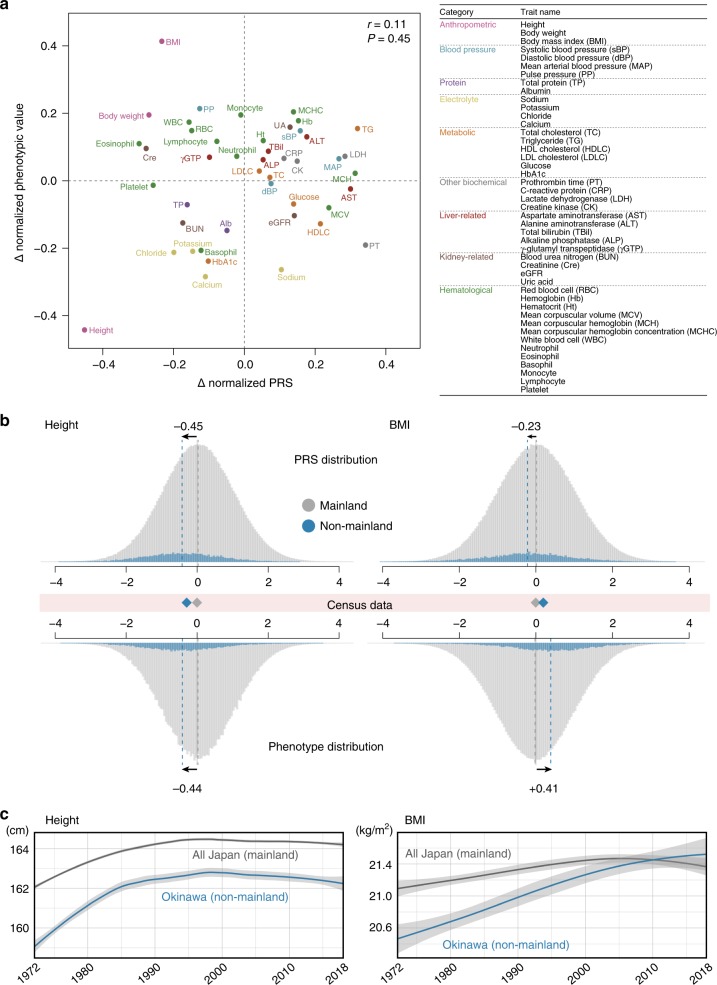
Polygenic risk score differentiations between mainland and non-mainland Japanese individuals. **a** Co-plot of the *Δ* normalized PRS and *Δ* normalized phenotypic value of 45 quantitative traits. The *Δ* normalized PRS (=normalized PRS in non-mainland−normalized PRS in mainland) is shown on the *x*-axis, and the *Δ* normalized phenotypic value (=normalized phenotypic value in non-mainland−normalized phenotypic value in mainland) is shown on the *y*-axis. Pearson’s correlation *r* and *P* value between the *Δ* normalized PRS and the *Δ* normalized phenotypic value are also described. The color of the dots represents the category of each trait. The right table shows the trait categories in color and the abbreviations of the traits. **b** Histograms of the PRS (top) and observed phenotypic value (bottom) for height (left) and BMI (right). In each panel, the distribution in the mainland is colored in gray, and that in non-mainland is colored in blue. The mean values of height and BMI retrieved from census data are shown in the middle, between the PRS and phenotypic histograms. The blue diamonds are the per-SD differences of height and BMI in non-mainland individuals. **c** Longitudinal census data of height (left) and BMI (right) in Japan. In each plot, the mean trait value of the general Japanese as a proxy of mainland (gray) and that of residents in Okinawa prefecture as a proxy of non-mainland (blue) are illustrated. The gray shadow indicates the 95% confidence interval.

We observed a considerable PRS difference between the non-mainlanders and the mainlanders; *Δ* normalized PRS ranged from −0.45 to 0.34 (*x*-axis in Fig. 5a). While the directions (i.e., signs of *Δ*) of the PRS deviations were concordant with those of the observed phenotypic deviations for more than half of the traits, we unexpectedly observed discordances of the directions (19 of 45 traits). For example, two representative polygenic traits, height, and body mass index (BMI), showed contrasting patterns (Fig. 5b). The negative PRS deviation of height in non-mainlanders was concordant with the phenotypic deviation and census data showing a shorter stature in non-mainlanders. This was in line with previous studies showing that the PRS deviations according to the regions had followed the actual height differences^34,35^. In contrast, the negative PRS deviation of BMI was discordant with the phenotypic deviation and census data showing a greater BMI in non-mainlanders. Overall, there was no correlation between the PRS deviation and the phenotypic deviation across the 45 traits (Pearson’s *r* = 0.11, *P* = 0.45). We note that no correlation was detected when we restricted the analysis to the traits with a variance explained by PRS exceeding 1% of the total trait variance (24/45 traits, Pearson’s *r* = 0.08, *P* = 0.72). This held true for the 22 binary traits (20 diseases and smoking/drinking habits; Supplementary Table 1). We again observed no correlation between the PRS difference and the difference in the prevalence of the diseases/habits (Pearson’s *r* = 0.26, *P* = 0.24, Supplementary Fig. 8).

We sought to tackle the challenging yet important question: what drives these systematic biases? First, we investigated the effect of the potential confounding factors that could bias PRS prediction. We assessed whether the PRS bias was associated with (a) heritability in the discovery GWAS, (b) the difference in GWAS heritability between the mainland and non-mainland populations, (c) variance explained by PRS, (d) the difference in variance explained by PRS between them, (e) differences in the distribution of confounding factors (i.e., age and sex) between them, and (f) the number of SNPs used in PRS calculations. Here, we defined the bias as the absolute difference between the PRS difference (=PRS_non-mainland_−PRS_mainland_) and the phenotypic difference (=PhenotypicValue_non-mainland_−PhenotypicValue_mainland_). We found that none of the potential confounding factors (a)–(f) were significantly correlated with the bias (Supplementary Table 2). We here note that these assessments should be validated further, given the small variances currently explained by PRSs in part of the investigated traits.

Next, from the phenotypic standpoint, we found a specific scenario regarding the smaller mean BMI PRS in non-mainlanders, despite the greater mean BMI observed in non-mainlanders. The longitudinal data of height and BMI in the Japanese population in the past 50 years revealed that the mean BMI in Okinawa prefecture (most of the non-mainland) exceeded that of the general Japanese population only a decade ago (Fig. 5c). Before that, the mean BMI in Okinawa prefecture had been less than that of the general Japanese population. If we assume that the BMI PRS predicts the BMI in the past one or two generations, the direction of the PRS difference is concordant with the phenotypic difference (smaller BMI in the non-mainland). Okinawa prefecture experienced a dietary habit change (i.e., westernization) after World War II, which might explain the rapid increase in BMI observed within Okinawa. This speculated scenario could be considered as a technical point in PRS prediction, especially when the phenotype is affected by relatively rapid environmental changes.

To summarize, we considered that the PRS distributions could be susceptible to the cryptic population substructures, even within a relatively homogeneous population. This bias might not be adjusted, even when the population structure was pre-detected and added as a covariate in GWASs. The reasons for this bias would be multifactorial and trait-dependent, which warrants further investigation. A careful assessment of the genetic and phenotypic architecture of complex traits is thus necessary when planning the risk stratification of individuals based on the relative rank of PRSs.

## Discussion

In this study, we demonstrated that PCA–UMAP, a novel non-linear dimensionality reduction method, could detect a fine and discrete population structure within the Japanese population at an unprecedentedly high resolution. The resolution reached high enough to uncover the genetic diversity among the adjacent insular subpopulations. The uncovered population structure was validated using the population genetics tools, which suggested the genetic diversity related to differences in demographic history. We finally assessed the differences in PRS distributions related to the substructures across a wide range of complex human traits. The deviation in PRSs did not always follow the phenotypic deviation, and these differences could not be corrected by including the identified population structures as one of the covariates. The reasons for this discrepancy were multifactorial, necessitating both genome and phenome-wise assessment in a trait-dependent manner.

We revealed that the genomic diversity within a population could be disentangled using the novel and strong analytical methodologies. Although we performed a proof-of-concept study in Japanese, British, Malaysian, and Arab populations, these approaches should be applied to diverse populations, given the vast increment in the ability to identify cryptic population structures. Our study also blows a whistle on the clinical applications of PRSs, which were more susceptible to subtle genetic differences than expected previously. Recently, there is a global action to expand genomic studies from European populations to diverse populations^36^. The expanded genetic studies yielded population-specific construction of PRSs^13^ and trans-ethnic PRS meta-analyses through worldwide collaborations^31^. That being said, however hard we may try to accommodate minute structures from worldwide populations into future genomic studies, it would be virtually unfeasible to perform well-powered GWASs for all subpopulations and for all the complex traits. Thus, we are in an emergent need for a practical methodology to derive PRSs to avoid the disadvantages of the underrepresented subpopulations.

In conclusion, with the novel dimensionality reduction methods in hand, we uncovered the finest-scale structure of the Japanese population. This structural diversity highlighted that PRS analysis still has a room for improvement to achieve truly individualized medicine.

## Methods

### Study participants, genotyping, and imputation

Individual genotype data were obtained from the BioBank Japan (BBJ) project^37,38^, a biobank that collaboratively collected DNA and serum samples from 12 medical institutions in Japan and recruited ~200,000 participants mainly of Japanese ancestry with a diagnosis of at least one of 47 diseases. We obtained informed consent from all participants according to the protocols approved by the ethical committee of the Institute of Medical Science, the University of Tokyo, before enrollment. This study was approved by the ethical committee of Osaka University Graduate School of Medicine.

We genotyped the individuals with the Illumina HumanOmniExpressExome BeadChip or a combination of the Illumina HumanOmniExpress and HumanExome BeadChips. Individuals who were identified as having a non-Japanese origin either by self-reporting or by PCA were excluded from the study^39^. Individuals with age < 18 or with low genotyping call rates (<98%) were additionally excluded^39,40^. For quality control (QC) of genotypes, we excluded variants that met any of the following criteria: (1) call rate < 99%, (2) *P* value for Hardy–Weinberg equilibrium < 1.0 × 10^−6^, and (3) number of heterozygotes < 5^39,40^. Genotype data from a total of 171,893 individuals of Japanese origin proceeded to the further analysis. Haplotype phasing on all 171,893 individuals was performed with Eagle (version 2.3)^41^ with default settings, to reduce the switch error rate. Any missing genotypes were imputed in the process of phasing.

### Dimensionality reduction to genotype data of Japanese

After removing genetically related individuals who were identified based on PI_HAT > 0.125 calculated by PLINK software^42^ and variants with a minor allele frequency < 0.01, we performed LD-pruning of the whole-genome variants, with an option “--indep-pairwise 50 5 0.2”. Pruned-in variants (*n*_variant_ = 91,551) from 169,719 individuals were used for the five dimensionality reduction methods (PCA, *t*-SNE, PCA–*t*-SNE, UMAP, and PCA–UMAP). First, we performed PCA on the pruned genotype matrix using EIGENSTRAT software^7^. The first two principal components were illustrated onto the two-dimensional space. Second, we performed *t*-SNE using multicoreTSNE package^43^ of the python software with default parameters. Third, we applied *t*-SNE to the first 50 principal components of the pruned genotypes with default parameters as PCA–*t*-SNE. Fourth, we performed UMAP on the genotype matrix using umap package^19^ of the python software (*n*_components_ = 2 and with default parameters). Fifth, we applied UMAP to the first 50 principal components of the pruned genotypes (*n*_components_ = 2 and default parameters) as PCA–UMAP. We annotated each of the individuals on the two-dimensional space by color, indicating the seven regions where the study individuals are recruited (namely, Hokkaido, Tohoku, Kanto-Koshinetsu, Chubu-Hokuriku, Kinki, Kyushu, and Okinawa). Individuals in Hokkaido-Ainu district, Hondo and Ryukyu were defined based on the result of PCA analysis as described in previous literature^15,16^. Individuals of the mainland and non-mainland clusters were classified based on the PCA–UMAP results (Fig. 2f). In addition to the genotypes of BBJ individuals, we merged them with those of East Asian individuals from the 1KGP phase3v5. The merged and pruned genotypes (*n*_total_ = 170,193) were used again as an input to the five dimensionality reduction methods.

Next, we applied PCA–UMAP to the genotype data from individuals within the mainland and non-mainland clusters. We annotated each of the individuals on the two-dimensional space by color, which denoted the above-mentioned regions from which the study individuals were recruited (the geographic distributions are depicted in Fig. 3b). As the secondary application of PCA–UMAP to individuals of the non-mainland cluster further separated them into distinct clusters, we defined them manually as the eight subclusters (i.e., Hokkaido-Ainu cluster 1, Kyushu clusters 2–6, and Okinawa clusters 7–8; Supplementary Fig. 4).

To clarify the positional relationship of each individual throughout the different dimensionality reduction methods, we used the Grimon (Graphical interface to visualize multi-omics networks; https://github.com/mkanai/grimon) software^44^ to visualize two dimensional plots from each of the dimensionality reduction methods onto three-dimensional parallel coordinates. We connected the corresponding individuals in the non-mainland cluster, and colored them according to the region information, as described above.

### fineSTRUCTURE analysis

We applied the ChromoPainter (CP) and fineSTRUCTURE (FS) (version 2) software on the subset of individuals representing the population substructures of Japan. We first randomly selected 200 individuals from the mainland populations and from each of the eight subclusters within the non-mainland populations. If a subcluster comprised <200 individuals, we adopted all of them. Thus, we selected a subset of mainland individuals (*n* = 200) and non-mainland individuals (*n* = 1,508). We used the phased haplotype data of these individuals with further QC of MAF > 0.05^25^ as an input for CP. The phased genotype files were converted into CP format and global switch and emission rates were estimated using CP’s expectation-maximization (EM) algorithm (10 iterations). Kerminen S. et al. verified that the EM estimates obtained using 24 individuals were not notably different from those obtained using larger samples of 238 individuals; thus, we used 102 of the 1708 individuals for the parameter estimation. CP was run using the estimated global parameters and the HapMap build 37 recombination map, which was converted into the CP format. Using CP’s chunkcounts output, FS was then run with the default options, without a predefined number of populations.

### Dimensionality reduction to worldwide genotype data

We additionally collected genotype data from UK Biobank^28^, MyEIRA study (Malaysian Epidemiological Investigation of rheumatoid Arthritis; Malaysian cohort to study the genetics of rheumatoid arthritis)^29^, and an Arab cohort (the Genetics of Rheumatoid Arthritis in Some Arab States Study)^30^.

The UK Biobank project is a population-based prospective cohort that recruited ~500,000 people aged between 40 and 69 years from 2006 to 2010 across the United Kingdom (https://www.ukbiobank.ac.uk/). Genotyping was conducted using either the Applied Biosystems UK BiLEVE Axiom Array or the Applied Biosystems UK Biobank Axiom Array. Genotypes were further imputed using a combination of the Haplotype Reference Consortium, UK10K, and 1000 Genomes Phase 3 reference panels. For the computational scalability, we randomly selected 13% of the genetically unrelated participants in the UK Biobank, as described elsewhere^28^. We collected the genotyped variants of those individuals from the imputed dataset. For QC, we excluded variants with a minor allele frequency < 0.05, *P*_HWE_ < 1 × 10^−6^, or INFO < 0.8. Then, we performed LD-pruning of the whole-genome variants, with an option “--indep-pairwise 50 5 0.1”. After LD-pruning, we had 54,293 individuals and 70,357 variants in total.

The MyEIRA (Malaysian Epidemiological Investigation of Rheumatoid Arthritis) is a population-based case-control study that recruited cases (rheumatoid arthritis) and controls in Peninsular Malaysia, which includes three major ethnic groups (i.e., Malays, Chinese, and Indians). The details of the MyEIRA have been described elsewhere^29,45^. The rheumatoid arthritis cases and controls were matched by age, sex, and residential area. All participants provided extensive information on socioeconomic background, lifestyle, life events as well as occupational exposures. Genotyping was conducted using an Illumina iSelect HD custom genotyping array (Immunochip, Illumina, Inc, San Diego, CA, USA). For QC, we excluded duplicate variants, indels, and variants with a minor allele frequency < 0.01 or *P*_HWE_ < 1 × 10^−6^. We imputed any missing variants using Beagle (version 5.0), and then performed LD-pruning of the whole-genome variants, with an option “--indep-pairwise 50 5 0.2”. After LD-pruning, we had 2831 individuals and 26,854 variants in total.

The individual information of the Arab cohort is described extensively elsewhere^30^. Participants were enrolled from five centers in Jordan, the Kingdom of Saudi Arabia, Lebanon, Qatar, and the United Arab Emirates. For QC, we excluded duplicate variants, indels, and variants with a minor allele frequency < 0.01 or *P*_HWE_ < 1 × 10^−6^. We imputed any missing variants using Beagle (version 5.0), and then performed LD-pruning of the whole-genome variants, with an option “--indep-pairwise 50 5 0.2”. After LD-pruning, we had 863 individuals and 89,503 variants in total.

For all three cohorts, the five dimensionality reduction methods were applied to the QCed and LD-pruned genotypes. The results were illustrated in the two-dimensional space and colored according to the self-reported ancestry in each of the cohorts.

### Ancestral analysis

ADMIXTURE (version 1.3.0) software was used to perform unsupervised estimation of an ancestral component of individuals from each of the subpopulations. We selected the individuals for this analysis based on the PCA–UMAP results. We randomly selected individuals in the mainland cluster (*n*_individual_ = 3000) and Okinawa cluster 7 (*n*_individual_ = 3000) to reduce the computational load. All individuals identified as belonging to other subclusters in the non-mainland cluster were included into the analysis. The variants used in the dimensionality reduction methods (*n*_variant_ = 91,551) were used as an input genotype for ADMIXTURE. ADMIXTURE was run with a *k* value (number of assumed ancestral components) ranging from 2 to 12. A representative result with *k* = 11 is illustrated in Fig. 3c, colored according to ancestral components and sorted based on the annotated subpopulations. All results with a *k* ranging from 2 to 12 are shown in Supplementary Fig. 7.

### Phylogenetic analysis

TreeMix software (version 1.13) was used to infer the patterns of population splits and mixtures of the worldwide and Japanese populations. We did not manually model the migration event, as we did not have concrete evidence for the migration and admixture events in Japan. We merged the genotype data of individuals from BBJ with those of the worldwide population in the 1KGP phase3 version 5. For the autosomal biallelic SNPs (*n*_variant_ = 481,954) which existed both in array genotype in BBJ and in whole genome sequence data in the 1KGP, we calculated the allele frequency stratified by the populations and subpopulations defined by PCA–UMAP. We then built the maximum-likelihood tree using blocks of 1000 SNPs to account for LD.

### PRS differentiation analysis

PRS should be constructed from the GWASs that enrolled the individuals from the matched population without overlap with the validation cohort. As BBJ is one of the largest genotyped biobanks of an East Asian population, we randomly split the study individuals into the discovery group (*n* = 84,908) and the validation group (*n* = 84,813). As we aimed to investigate PRS differences between the mainland and non-mainland clusters, we set two cohorts to have approximately the same number of individuals from those clusters. We conducted GWASs of the 45 quantitative traits and 22 case-control binary traits in the discovery group. The detailed information of phenotypes of individuals in the discovery group is summarized in Supplementary Table 1. To account for the population stratification, we included sex, age, 20 principal components, and whether an individual was included in the mainland cluster or the non-mainland cluster as covariates. The study-specific exclusion criteria and covariates for GWASs of the quantitative traits are described in Supplementary Data 1.

Using the summary statistics of these 67 GWASs, we constructed PRSs using a thresholding and clumping method. For *P* value thresholding, we adopted a relatively conservative *P* value threshold of 5.0 × 10^−6^, since PRSs based on large numbers of SNPs below genome-wide significance are susceptible to biases due to the uncorrected population structure^46^. LD clumping was performed using the PLINK software (version 1.90), so that the variants at least 1 Mb apart and with LD *r*^2^ < 0.1 were selected. Individual scores were created by adding the dosage of the risk alleles at each variant and then multiplying the sum by the effect size from the discovery group. The derived PRSs were rank-normalized within the validation group to remove outlier effects. The discovery GWAS heritability (hg), LD score regression (LDSC) intercept^47^, and the variance explained by the PRS in the validation group are described in Supplementary Table 3. The variance explained by the PRS was obtained by subtracting the adjusted *R*-squared in a nested linear regression model (phenotype~covariates) from that in a full linear regression model (phenotype~PRS + covariates). The PRS differentiation (*Δ* normalized PRS) of each phenotype was obtained by subtracting the mean of the normalized PRS of the mainland individuals from those of the non-mainland individuals as in the following equation:

1 $${\Delta {\mathrm{{{PRS}}}}_{{\mathrm{{{normalized}}}}}} =	 \,\,\mathrm {mean}\left( {{\mathrm{{{PRS}}}}_{{\mathrm{{{normalized}}}}}\,{\mathrm{{{in}}}}\,{\mathrm{{{nonmainland}}}}} \right) \\ 	-{\mathrm{{mean}}}\left( {{\mathrm{{{PRS}}}}_{{{\mathrm{{{normalized}}}}}}\,{\mathrm{{{in}}}}\,{\mathrm{{{mainland}}}}} \right)$$

The phenotype differentiation (*Δ* normalized phenotypic value) was obtained by subtracting the mean of the normalized phenotype of mainland individuals from that of non-mainland individuals as in the following equation:

2 $$\Delta {\mathrm{PhenotypicValue}}_{{\mathrm{normalized}}}=	 \,\,{\mathrm{{mean}}}\left( {{\mathrm{PhenotypicValue}}_{{\mathrm{normalized}}}\,{\mathrm{in}}\,{\mathrm{nonmainland}}} \right) \\ \,	- {\mathrm{{mean}}}\left( {{\mathrm{PhenotypicValue}}_{{\mathrm{normalized}}}\,{\mathrm{in}}\,{\mathrm{mainland}}} \right)$$

Phenotype normalization was performed by rank-normalizing the residuals of phenotypes regressed on sex, age and age^2^ as covariates. For height and BMI, we illustrated the histograms of the normalized PRS of mainland individuals (gray) and of non-mainland individuals (blue), and the histograms of normalized phenotype of mainland individuals (gray) and of non-mainland individuals (blue). To validate the phenotypic differentiations externally, we obtained height and BMI statistics within each prefecture in Japan from the census data provided by the Japanese government in 2018 and 2017, respectively (e-Stat). The mean phenotypic value is shown on the histograms to indicate the per SD phenotypic difference between mainland individuals and non-mainland individuals. As the census data was on prefecture-basis, the mean phenotypic value in mainland individuals was represented by that for the general Japanese population. The mean phenotypic value in non-mainland individuals was represented by that in residents of Okinawa prefecture, which comprised the majority of the non-mainland cluster.

### Correlation between confounding factors and the bias in PRS

We selected six potential confounding factors that might potentially cause a bias in polygenic risk predictions. The details of this analysis are also described in Supplementary Table 2.

- GWAS heritability in the discovery GWAS: we obtained the heritability estimations of each trait in the discovery GWAS, calculated using the LDSC software.
- Differences in GWAS heritability between the mainland and non-mainland populations: we calculated the GWAS heritability within the mainland and non-mainland populations using the GCTA software^48^ with a randomly selected same number of individuals. As the number of recorded phenotypes was always lower in the non-mainland population than in the mainland population, we adopted the number of effective phenotypes in the non-mainland population for random selection among the mainland population. Then, we obtained the absolute differences between the heritability values (mainland vs. non-mainland).
- Variance explained by PRS in a whole cohort: we obtained the variance explained by PRS in the validation cohort per trait, as described in the previous section.
- Differences in variance explained by PRS between the mainland and non-mainland populations: we calculated the variance explained by PRS per trait, separately in mainland and non-mainland individuals. We then obtained the absolute differences between the variances (mainland vs. non-mainland).
- Differences in the distribution of confounding factors between the mainland and non-mainland populations: we obtained the age distribution and the sex composition between, separately for the mainland and non-mainland populations.
- The number of SNPs used in PRS calculations: we obtained the number of SNPs used in the calculation of PRS (weighted allelic sum of these SNPs) per trait.

Next, we defined the bias in per-trait PRS as in the following equation:

3 $${\mathrm{BIAS}}_{{\mathrm{trait}}} =	 \,\, \left| \left( {{\mathrm{PRS}}_{{\mathrm{nonmainland}},{\mathrm{trait}}} - {\mathrm{PRS}}_{{\mathrm{mainland}},{\mathrm{trait}}}} \right) \right. \\ \,	- \left. \left( {{\mathrm{PhenotypicValue}}_{{\mathrm{nonmainland}},{\mathrm{trait}}} - {\mathrm{PhenotypicValue}}_{{\mathrm{mainland}},{\mathrm{trait}}}} \right) \right|$$

With the exception of (e), we performed a correlation test between this bias and the quantitative value of potential confounding factors across 45 traits, and obtained Pearson’s *r* and *P* values as in the following equation:

4 $${\mathrm{{BIAS}}}\sim {\mathrm{CONFOUNDING}}_{\left( {\mathrm{i}} \right)},\,{\mathrm{{where}}}\,\left( {\mathrm{i}} \right)\,{\mathrm{{is}}}\,\left( {\mathrm{a}} \right),\,\left( {\mathrm{b}} \right),\,\left( {\mathrm{c}} \right),\,\left( {\mathrm{d}} \right),\,{\mathrm{{or}}}\,\left( {\mathrm{f}} \right)$$

### Longitudinal data of height and BMI

We collected the longitudinal census data of height and BMI from annual school health checkup data across Japan and according to the prefectures of this country (e-Stat). Starting in 1972 (when the Okinawa prefecture returned to the Japanese government from US occupation and census data collection started), every other year, we collected the mean height and BMI of students aged 17 years in Japan as a proxy of the mainland, and of those in Okinawa prefecture as a proxy of the non-mainland. As the data were generated separately for males and females, we used the average of the values in males and those in females. Then, for each dataset, we plotted a regression line with 95% confidence interval based on local polynomial regression fitting, by using the geom_smooth() function in the ggplot2 package of the R statistical software.

### Reporting summary

Further information on research design is available in the Nature Research Reporting Summary linked to this article.

## Supplementary information

Supplementary Information

Supplementary Data 1

Reporting Summary

Description of Additional Supplementary Files

## Data Availability

The genotype data of BioBank Japan used in this study are available from the Japanese Genotype–phenotype Archive (JGA; http://trace.ddbj.nig.ac.jp/jga/index_e.html) with accession code JGAD00000000123 [https://ddbj.nig.ac.jp/jga/viewer/view/study/JGAS00000000114]. The GWAS summary statistics of BioBank Japan are available at the National Bioscience Database Center (NBDC) Human Database with the accession code hum0014 [https://humandbs.biosciencedbc.jp/en/hum0014-v18]. UK Biobank analysis was conducted via the application 31063. All other data are contained in the article file and its supplementary information or available upon request.

## Code availability

We provide the custom scripts that we used to perform five dimensionality reduction methods on genotype at https://github.com/saorisakaue/Genotype-dimensionality-reduction. We also used the publicly available softwares, which were listed and described in the Methods section in our manuscript.
