# Supplementary material for: Dimensionality reduction reveals fine-scale structure in the Japanese population with consequences for polygenic risk prediction
Source: Nat Commun. 2020 Mar 26;11:1569. doi: 10.1038/s41467-020-15194-z (PMC7099015; doi:10.1038/s41467-020-15194-z)
Supplement: Supplementary file 1 — Supplementary Information [file 41467_2020_15194_MOESM1_ESM.pdf]

Supplementary Information for

Dimensionality reduction reveals fine-scale structure in the Japanese population with consequences for polygenic risk prediction.

Sakaue et al.

Table of Contents:

Page 3	**Supplementary Figure 1**
Page 4	**Supplementary Figure 2**
Page 5	**Supplementary Figure 3**
Page 6	**Supplementary Figure 4**
Page 7	**Supplementary Figure 5**
Page 8	**Supplementary Figure 6**
Page 9	**Supplementary Figure 7**
Page 10	**Supplementary Figure 8**
Page 11	**Supplementary Table 1**
Page 12	**Supplementary Table 2**
Page 13	**Supplementary Table 3**

Supplementary Data 1 is provided by a separate excel file.

Supplementary Figure 1.

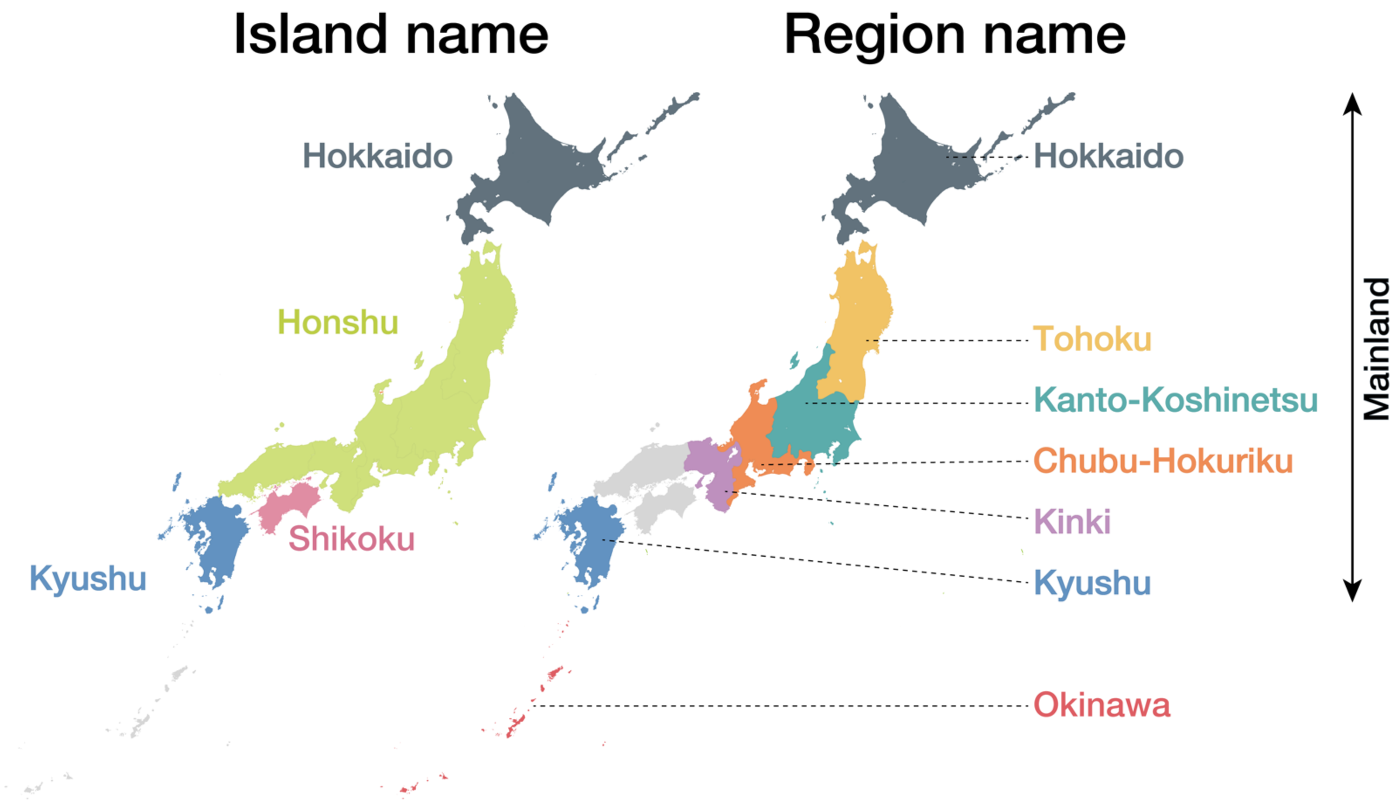

Supplementary Figure 1. The geographic names of Japan.

Left panel shows that the mainland of Japan consists of four major islands (i.e., “Hokkaido”, “Honshu”, “Shikoku”, and “Kyushu”). Right panel shows seven regional information of recruitment centers in our study. Of them, the mainland includes six regions (i.e., “Hokkaido”, “Tohoku”, “Kanto-Koshinetsu”, “Chubu-Hokuriku”, “Kinki”, and “Kyushu”).

Supplementary Figure 2.

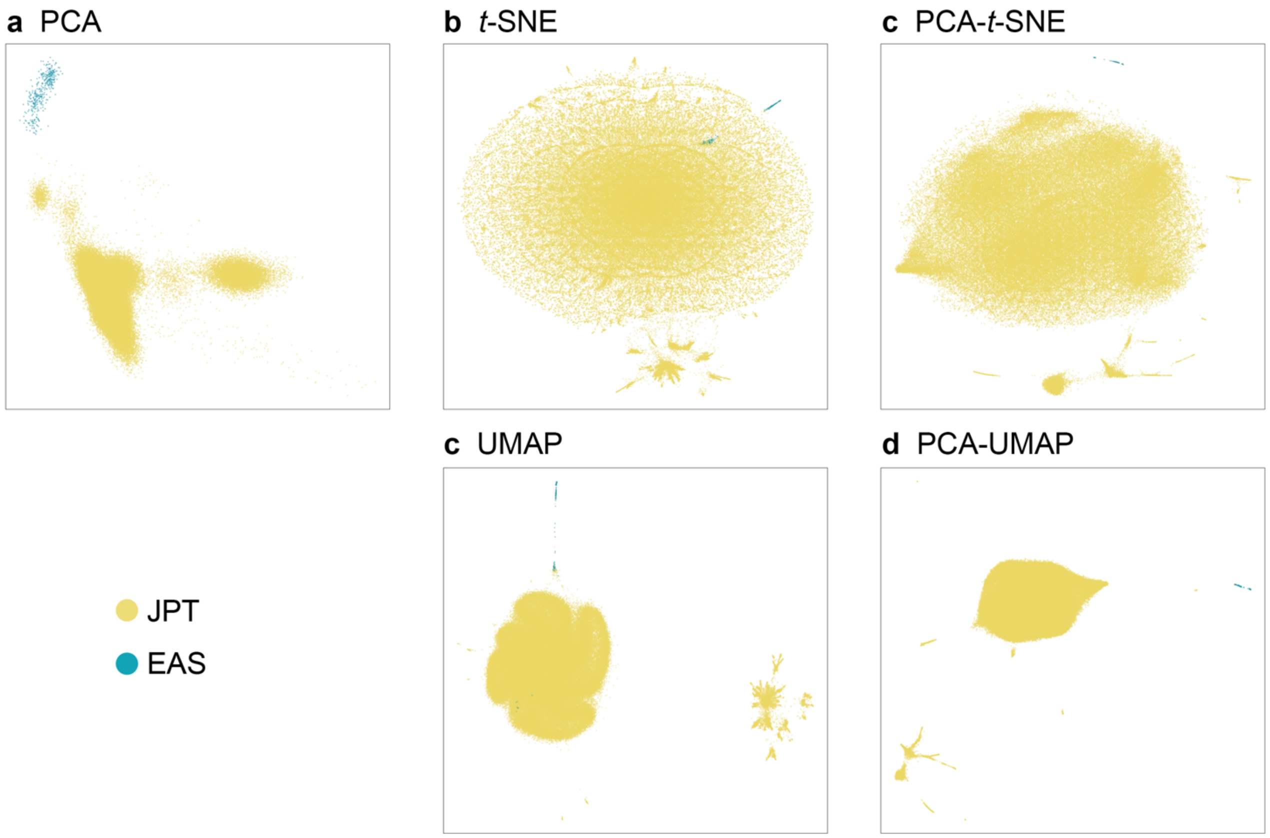

Supplementary Figure 2. Dimensionality reduction methods applied to genotype data of Japanese and other East Asian populations.

Shown are two-dimensional illustrations of genotype data of the Japanese population in BioBank Japan (JPT) and other East Asian populations from the 1KGP (EAS). Each plot is colored in yellow (JPT) or green (EAS) according to the population. Other East Asian populations consisted of Han Chinese, Southern Han Chinese, Chinese Dai and Kihn.

Supplementary Figure 3.

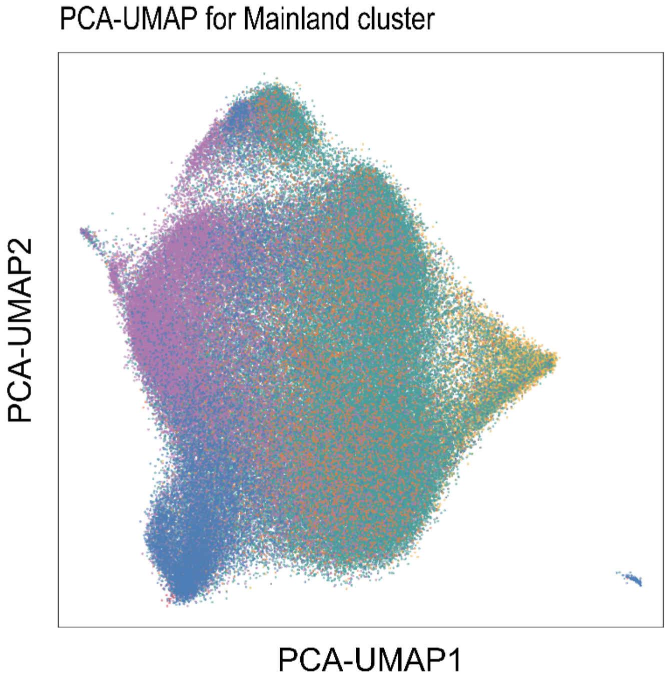

Supplementary Figure 3. Secondary PCA-UMAP applied for mainland individuals defined by the primary PCA-UMAP.

The color of individual points indicates the region where a given study individual was recruited. The definition of the regions and colors is the same as in **Figure 2**.

Supplementary Figure 4.

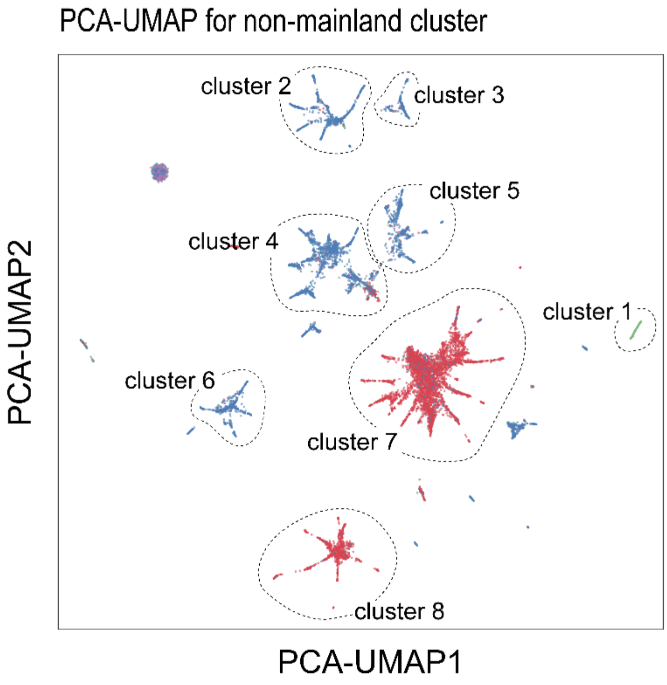

Supplementary Figure 4. Secondary PCA-UMAP applied for non-mainland individuals defined by the primary PCA-UMAP.

The definition of subclusters according to the result of the secondary PCA-UMAP analysis for non-mainland individuals is shown by the dotted lines. The color of individual points indicates the region where a given study individual was recruited. The definition of the regions and colors is the same as in **Figure 2**.

Supplementary Figure 5

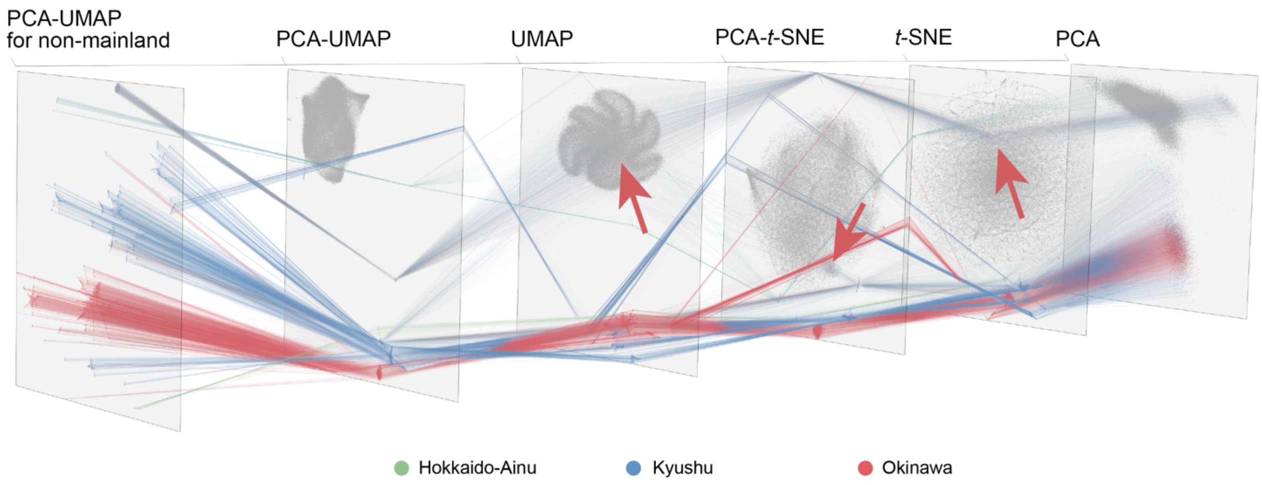

Supplementary Figure 5. The three-dimensional paralleled illustration showing the connection between the dimensionality reduction methods.

The results of five dimensionality reduction methods (PCA, *t*-SNE, PCA-*t*-SNE, UMAP and PCA-UMAP for all individuals, and PCA-UMAP for non-mainland individuals) are shown in transitional layers from right to left. Individuals in the non-mainland cluster are colored according to the regions in the same way as in **Figure 2** and **3**. A red arrow denotes where the individual plot of the non-mainland population, which was defined in PCA-UMAP, fell into the largest cluster (i.e., the mainland cluster) in other dimensionality reduction methods.

Supplementary Figure 6

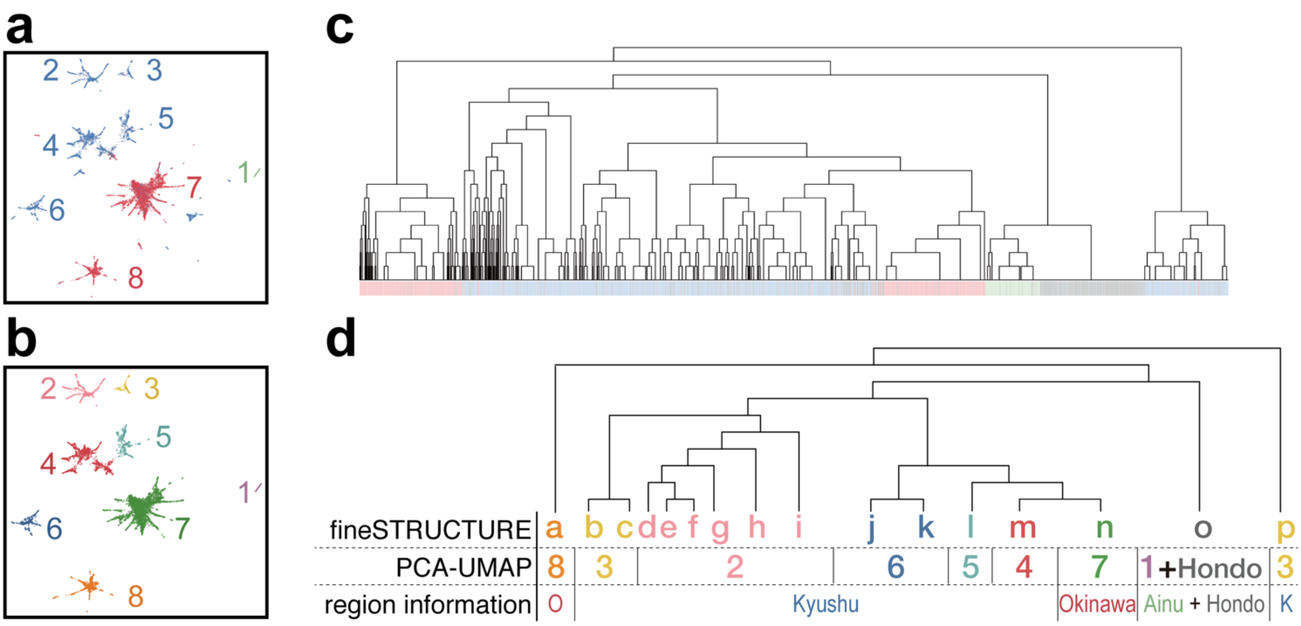

Supplementary Figure 6. Correspondence between fineSTRUCTURE and regional information or PCA-UMAP results.

(a) Secondary PCA-UMAP results to non-mainland individuals, annotated and colored according to the regional information of participants. A plot in green indicates an individual of Ainu, that in blue indicates an individual of Kyushu, and that in red indicates an individual of Okinawa (as in **Figure 3a**). **(b)** Secondary PCA-UMAP results to non-mainland individuals, annotated and colored according to the subclusters defined in **Supplementary Figure 4**. **(c)** A clustering result of fineSTRUCTURE, where each individual is annotated and colored according to the subclusters defined by regional information of participants (as in panel **(a)**). **(d)** A truncated hierarchical clustering of fineSTRUCTURE at the level of 16 clusters, and a correspondence across fineSTRUCTURE-defined clusters (top), PCA-UMAP-defined subclusters (middle), and the regional information of recruitment centers (bottom). O; an abbreviation for Okinawa. K; an abbreviation for Kyushu.

Supplementary Figure 7.

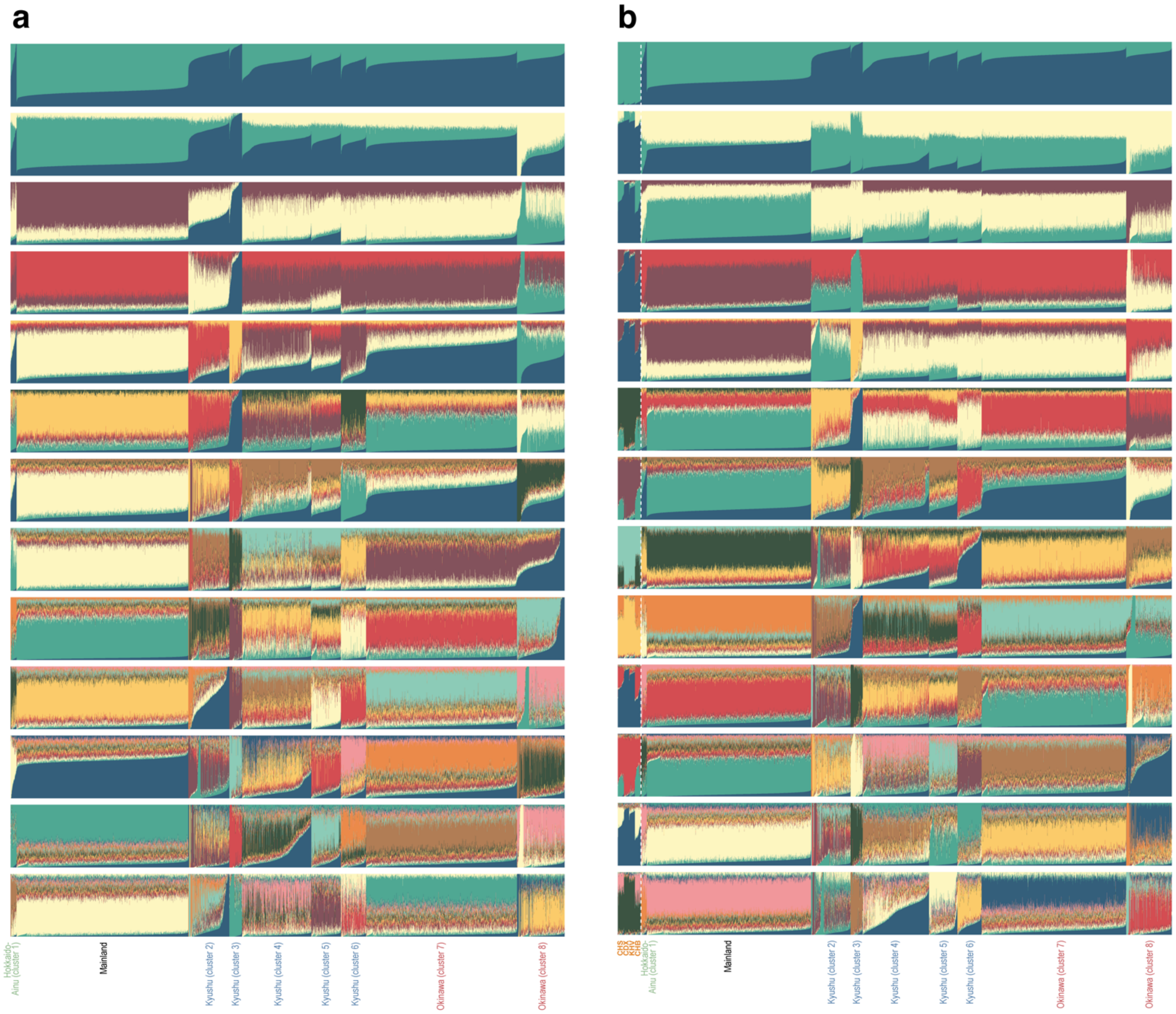

Supplementary Figure 7. ADMIXTURE analyses with varying number of ancestral components.

The unsupervised maximum-likelihood estimation under a model with 2 to 14 ancestral components (from top to bottom). **(a)** The result for BioBank Japan individuals. **(b)** The result for BioBank Japan individuals merged with other East Asian populations in 1KGP (i.e., Southern Han Chinese [CHS], Chinese Dai in Xishuangbanna [CDX], Kinh in Ho Chi Minh City [KHV], Han Chinese in Beijing [CHB]).

Supplementary Figure 8.

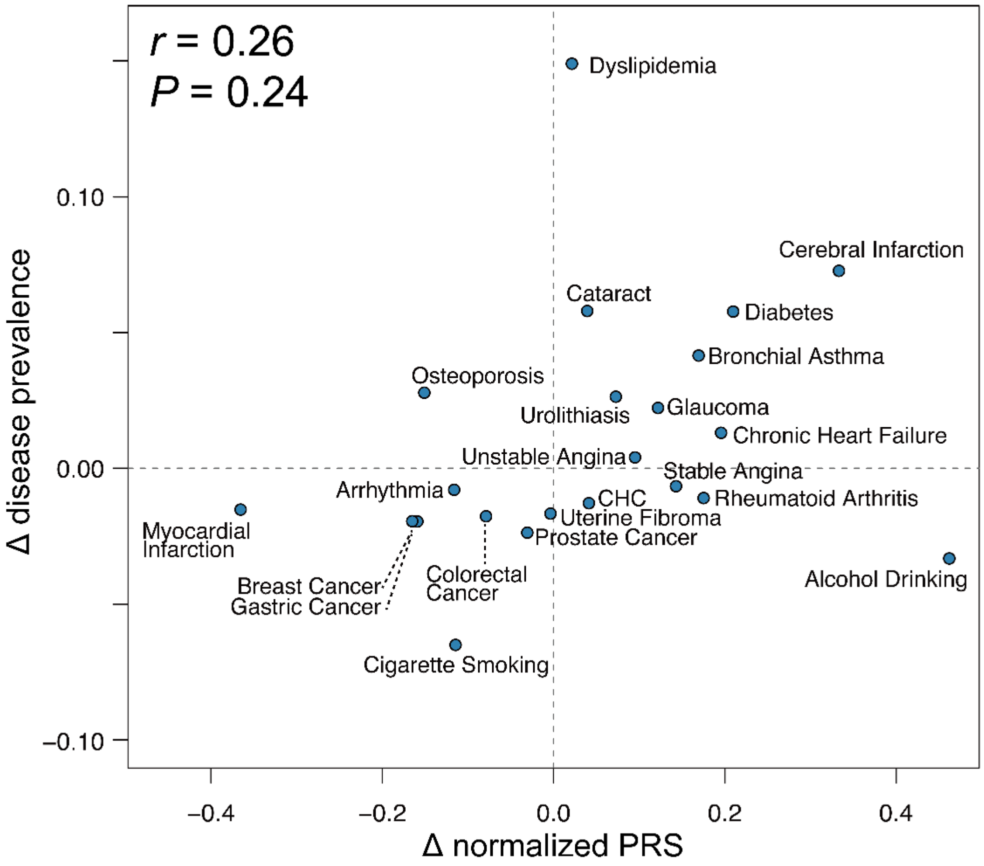

Supplementary Figure 8. The Δ normalized PRS and Δ disease prevalence of 22 binary traits.

Δ normalized PRS (= normalized PRS in non-mainland - normalized PRS in mainland) is shown on the x-axis, and *Δ* disease prevalence (= disease prevalence in non-mainland - disease prevalence in mainland) is shown on the y-axis. Pearson's correlation *r* and *P* value between *Δ* normalized PRS and *Δ* disease prevalence are also described.

Supplementary Table 1. Phenotype summary in the discovery GWASs.

Quantitative traits

Trait name	No. no missing value	Mean	Median	Standard deviation	Minimum	Maximum	Unit
Height	78,558	160	160	9.03	117	198	cm
Body weight (BW)	78,756	59.4	58.5	11.5	23	110	kg
Body mass index (BMI)	78,107	23.3	23	3.62	11.4	46.2	kg m^{-2}
Systolic blood pressure (sBP)	69,270	132	130	18.3	70	213	mmHg
Diastolic blood pressure (dBP)	69,235	77.5	79	11.5	35	125	mmHg
Mean arterial blood pressure (MAP)	69,173	95.8	96	12.5	51	150	mmHg
Pulse pressure (PP)	69,173	54.7	54	13.8	10	145	mmHg
Total protein	58,599	7.09	7.1	0.569	4.7	9.5	g dL^{-1}
Albumin	53,043	4.2	4.3	0.45	2.1	6.1	g dL^{-1}
Uric acid	59,968	5.43	5.3	1.53	0.3	12.3	mg dL^{-1}
Sodium	64,021	141	141	2.72	128	154	mEq L^{-1}
Potassium	66,644	4.24	4.2	0.406	2.6	5.9	mEq L^{-1}
Chloride	63,186	104	105	2.88	94.9	114	mEq L^{-1}
Calcium	39,035	9.18	9.2	0.527	6.8	11.5	mg dL^{-1}
Total cholesterol (TC)	64,650	208	205	45.7	20	469	mg dL^{-1}
Triglyceride (TG)	53,954	140	116	96.8	9.4	1490	mg dL^{-1}
HDL cholesterol (HDL-C)	35,620	55	53	15.7	0.1	123	mg dL^{-1}
LDL cholesterol (LDL-C)	36,766	138	128	52.9	0	496	mg dL^{-1}
Blood sugar	47,539	109	102	26.9	10.2	254	mg dL^{-1}
HbA1c	21,737	5.55	5.4	0.764	2.96	10.7	%
Prothrombin time	24,048	11.8	11.6	1.28	8.2	18.9	sec
C-reactive protein	38,399	0.257	0.12	0.304	0	1.7	mg dL^{-1}
AST	70,599	24.2	22	11	5	105	IU L^{-1}
ALT	70,973	24.6	19	19.4	1.6	256	IU L^{-1}
Total Bilirubin	56,832	0.626	0.6	0.351	0.05	6.2	mg dL^{-1}
LDH	65,995	233	198	107	45	993	IU L^{-1}
Alkaline phosphatase	54,104	241	225	97.8	45	1160	IU L^{-1}
γ-GTP	63,836	45.8	27	62.2	1	905	IU L^{-1}
Creatine kinase	55,094	112	90	92.7	7.3	1140	IU L^{-1}
Blood urea nitrogen	70,769	15.7	15	5.38	3.6	64	mg dL^{-1}
Creatinine	72,278	0.786	0.74	0.252	0.2	2.97	mg dL^{-1}
eGFR	72,280	73.6	72.5	20.9	20	250	mL min^{-1} 1.73 m^{-2}
Red blood cell (RBC)	68,406	436	438	54.3	187	665	10^4 per μL
Hb	68,024	13.5	13.5	1.72	5.5	20	g dL^{-1}
Ht	68,197	40.3	40.5	4.77	18.7	61.7	%
MCV	58,184	92.8	92.9	5.26	70.4	115	fL
MCH	57,479	31	31.1	2.03	22.6	39.4	pg
MCHC	60,679	33.4	33.4	1.08	28.6	38.2	%
White blood cell (WBC)	69,000	6240	5900	2080	1450	23800	per μL
Neutrophil	37,926	3750	3420	1680	572	20100	per μL
Eosinophil	41,926	197	140	215	2.7	6590	per μL
Basophil	39,008	40.9	31.8	39.4	0.987	994	per μL
Monocyte	42,944	385	350	190	44.4	2670	per μL
Lymphocyte	43,093	1820	1720	762	250	10500	per μL
Platelet (PLT)	66,096	22.9	22	7.16	5.8	83.1	10^4 per μL

Binary Case Control Traits

Trait name	No. case	No. control
Arrhythmia	7,043	76,166
Chronic Heart Failure	3,215	79,994
Myocardial Infarction	5,711	77,498
Stable Angina	7,180	76,029
Unstable Angina	1,919	81,290
Cerebral Infarction	7,180	76,029
Diabetes	17,242	65,967
Dyslipidemia	19,025	64,184
Breast Cancer	2,377	35,929
Colorectal Cancer	2,870	80,339
Gastric Cancer	2,745	80,464
Prostate Cancer	2,201	42,702
Bronchial Asthma	3,453	79,756
Rheumatoid Arthritis	1,870	81,339
Chronic Hepatitis C	2,560	80,649
Cataract	8,504	74,705
Glaucoma	1,992	81,217
Osteoporosis	2,763	80,446
Urolithiasis	2,779	80,430
Uterine Fibroma	2,619	35,687
Ever-drink-alcohol	41,558	40,362
Ever-smoke-cigarette	41,336	40,724

Supplementary Table 2. Potential confounding factors in PRS prediction and their correlation with the observed PRS biases.

	Investigated factor	correlation *r*	correlation *P*	Descriptions
**a**	GWAS heritability	0.17	0.24	We assessed whether the lower the heritability (i.e. less predictive ability in PRS), the larger the bias in PRS.
**b**	Difference in GWAS heritability	-0.30	0.044	We calculated GWAS heritability within mainland and non-mainland population by GCTA software with the randomly selected same number of samples. We assessed whether the larger the difference in heritability, the larger the bias in PRS.
**c**	Variance explained by PRS	0.030	0.85	We assessed whether the lower the variance explained (i.e. less predictive ability in PRS), the larger the bias.
**d**	Difference in variance explained by PRS	0.13	0.38	We assessed whether the larger the difference in variance explained, the larger the bias.
**e**	Differences in potential confounding factors	-	-	According to the recent preprint (Mostafavi H et al. ***bioRxiv*** 2019), the prediction accuracy of PRSs depended on characteristics such as the age or sex composition of the individuals in GWAS even within a single ancestry (white British people in UK Biobank). Thus, we assessed whether there was difference in age and sex composition between mainland and non-mainland population. We observed that age and sex composition was mostly comparable between them (Median age; 65 vs 66 and Fraction of females; 45.6% vs 47.5%, respectively).
**f**	Number of SNPs used in PRS	-0.014	0.93	We assessed whether the more the number of SNPs in PRS (i.e. suceptible to genetic drift), the larger the bias in PRS.

Correlation *r* and *P* are calculated from Pearson's correlation test. Details are described in **Methods**.

Supplementary Table 3. Summary results of GWASs and PRSs of quantitative traits.

Trait name	Observed scale h^2 (SE)	Lambda GC	Mean Chi^2	Intercept (SE)	Ratio (SE)	No. SNPs in PRS	Explained variance by PRS
Height	0.338 (0.019)	1.3615	1.6312	1.0631 (0.0134)	0.1 (0.0213)	492	0.0360
Body weight (BW)	0.1737 (0.0101)	1.2332	1.2937	1.0138 (0.0088)	0.047 (0.0301)	79	0.0126
Body mass index (BMI)	0.1941 (0.0109)	1.2731	1.3469	1.0321 (0.0092)	0.0927 (0.0267)	127	0.0106
Systolic blood pressure (sBP)	0.0578 (0.0082)	1.0864	1.0998	1.0154 (0.0074)	0.1546 (0.0742)	36	0.00197
Diastolic blood pressure (dBP)	0.0487 (0.0077)	1.0679	1.0762	1.0073 (0.0072)	0.0956 (0.0941)	19	0.000585
Mean arterial blood pressure (MAP)	0.0535 (0.0082)	1.0802	1.0902	1.014 (0.0076)	0.1549 (0.0842)	22	0.00200
Pulse pressure (PP)	0.0428 (0.0075)	1.0649	1.0765	1.0143 (0.0073)	0.1863 (0.0953)	28	0.000687
Total protein	0.0873 (0.0117)	1.105	1.1294	1.0219 (0.0083)	0.1694 (0.0639)	69	0.0126
Albumin	0.0603 (0.0104)	1.0618	1.0743	1.0071 (0.0076)	0.0957 (0.103)	27	0.00322
Uric acid	0.141 (0.0377)	1.0833	1.2036	0.9997 (0.0075)	< 0	180	0.0378
Sodium	0.0683 (0.0091)	1.0772	1.0903	0.9969 (0.0081)	< 0	25	0.00163
Potassium	0.0574 (0.0107)	1.0895	1.0951	1.0141 (0.0085)	0.1478 (0.0895)	25	0.00148
Chloride	0.0658 (0.0088)	1.0864	1.1032	1.0164 (0.0078)	0.1589 (0.0753)	40	0.00224
Calcium	0.042 (0.0157)	1.0557	1.0524	1.0227 (0.0071)	0.4338 (0.1354)	29	0.00275
Total cholesterol (TC)	0.084 (0.0127)	1.0772	1.1336	1.0192 (0.0093)	0.1435 (0.0699)	127	0.0316
Triglyceride (TG)	0.0993 (0.0296)	1.0618	1.1343	1.0236 (0.0087)	0.1758 (0.0646)	121	0.0332
HDL cholesterol (HDL-C)	0.1515 (0.0255)	1.0864	1.1484	1.0391 (0.009)	0.2635 (0.0607)	150	0.0780
LDL cholesterol (LDL-C)	0.0748 (0.0167)	1.0405	1.0656	1.0073 (0.0081)	0.1115 (0.1235)	50	0.0246
Blood sugar	0.0366 (0.0111)	1.0466	1.0574	1.0215 (0.0074)	0.3742 (0.1284)	42	0.00616
HbA1c	0.1325 (0.0266)	1.0466	1.0735	1.0149 (0.0076)	0.2027 (0.1029)	36	0.0133
Prothrombin time	0.0426 (0.0246)	1.0285	1.038	1.0159 (0.0077)	0.4189 (0.2035)	31	0.0178
C-reactive protein	0.0216 (0.0126)	1.0496	1.0567	1.0397 (0.0071)	0.7003 (0.1254)	38	0.00268
AST	0.0661 (0.0105)	1.0833	1.1128	1.0156 (0.0084)	0.1385 (0.0743)	59	0.00856
ALT	0.0578 (0.0107)	1.0649	1.0976	1.0095 (0.0084)	0.0975 (0.0857)	56	0.00499
Total Bilirubin	0.0622 (0.0285)	1.0496	1.098	1.0237 (0.007)	0.2417 (0.0713)	101	0.0544
LDH	0.0366 (0.0074)	1.0833	1.0815	1.0324 (0.0077)	0.3973 (0.0942)	60	0.00958
Alkaline phosphatase	0.1226 (0.0426)	1.0988	1.1695	1.0403 (0.0101)	0.2375 (0.0596)	152	0.0504
γ-GTP	0.0819 (0.0144)	1.0802	1.1278	1.0219 (0.0079)	0.1712 (0.0617)	92	0.0103
Creatine kinase	0.0978 (0.012)	1.0926	1.1274	1.0158 (0.0081)	0.124 (0.0636)	94	0.0101
Blood urea nitrogen	0.0708 (0.0091)	1.1082	1.1381	1.0346 (0.008)	0.2504 (0.058)	48	0.00719
Creatinine	0.1038 (0.0109)	1.1459	1.1926	1.0331 (0.0088)	0.1716 (0.0455)	111	0.00647
eGFR	0.1151 (0.0111)	1.1459	1.2089	1.0318 (0.0094)	0.1523 (0.045)	101	0.00953
Red blood cell (RBC)	0.1271 (0.0144)	1.1459	1.2183	1.0338 (0.0102)	0.1548 (0.0467)	152	0.0253
Hb	0.0778 (0.0098)	1.0988	1.1275	1.016 (0.0086)	0.1259 (0.0671)	64	0.00681
Ht	0.0813 (0.0094)	1.105	1.1337	1.0157 (0.0089)	0.1177 (0.0665)	63	0.00804
MCV	0.1985 (0.0232)	1.1333	1.266	1.0222 (0.0106)	0.0833 (0.0398)	234	0.0636
MCH	0.1746 (0.023)	1.1113	1.2359	1.0245 (0.0103)	0.1038 (0.0435)	222	0.0618
MCHC	0.0512 (0.0124)	1.0557	1.0967	1.0327 (0.0087)	0.3376 (0.0899)	93	0.0231
White blood cell (WBC)	0.1165 (0.0119)	1.1459	1.1966	1.0314 (0.0101)	0.1597 (0.0514)	103	0.0140
Neutrophil	0.0952 (0.0175)	1.0741	1.0939	1.0194 (0.0088)	0.2071 (0.0937)	52	0.0110
Eosinophil	0.0618 (0.0155)	1.0618	1.0852	1.0312 (0.0073)	0.3662 (0.0859)	59	0.00595
Basophil	0.0933 (0.0173)	1.0741	1.1031	1.0299 (0.0083)	0.2904 (0.081)	65	0.0104
Monocyte	0.0742 (0.0144)	1.071	1.0997	1.0346 (0.0082)	0.3474 (0.0823)	70	0.0159
Lymphocyte	0.0749 (0.0124)	1.0679	1.0804	1.0128 (0.0081)	0.1598 (0.1007)	41	0.00584
Platelet (PLT)	0.1664 (0.0159)	1.1523	1.2567	1.0284 (0.0084)	0.1105 (0.0328)	227	0.0383

SE; standard error.
