# Supplementary material for: Dimensionality reduction reveals fine-scale structure in the Japanese population with consequences for polygenic risk prediction
Source: Nat Commun. 2020 Mar 26;11:1569. doi: 10.1038/s41467-020-15194-z (PMC7099015; doi:10.1038/s41467-020-15194-z)
Supplement: Supplementary file 4 — Description of Additional Supplementary Files [file 41467_2020_15194_MOESM4_ESM.pdf]

Title: Supplementary Data 1
Description: Study specific exclusion criteria and covariates used for the quantitative trait analysis
